# Structure of archaerhodopsin-2 at 1.8 Å resolution

**DOI:** 10.1107/S1399004714017313

**Published:** 2014-09-27

**Authors:** Tsutomu Kouyama, Ryudo Fujii, Soun Kanada, Taichi Nakanishi, Siu Kit Chan, Midori Murakami

**Affiliations:** a Department of Physics, Graduate School of Science, Nagoya University, Nagoya, Japan; b RIKEN Harima Institute/SPring-8, 1-1-1 Kouto, Mikazuki, Sayo, Hyogo, Japan

**Keywords:** archaerhodopsin-2, light-driven proton pump

## Abstract

Archaerhodopsin-2 (aR2), the sole protein found in the claret membrane of *Halorubrum* sp. Aus-2, functions as a light-driven proton pump. In this study, structural analysis of aR2 was performed using a novel three-dimensional crystal prepared by the successive fusion of claret membranes. The crystal is made up of stacked membranes, in each of which aR2 trimers are arranged on a hexagonal lattice. This lattice structure resembles that found in the purple membrane of *H. salinarum*, except that lipid molecules trapped within the trimeric structure are not distributed with perfect threefold symmetry. Nonetheless, diffraction data at 1.8 Å resolution provide accurate structural information about functionally important residues. It is shown that two glutamates in the proton-release channel form a paired structure that is maintained by a low-barrier hydrogen bond. Although the structure of the proton-release pathway is highly conserved among proton-pumping archaeal rhodopsins, aR2 possesses the following peculiar structural features: (i) the motional freedom of the tryptophan residue that makes contact with the C13 methyl group of retinal is restricted, affecting the formation/decay kinetics of the L state, and (ii) the N-terminal polypeptide folds into an Ω-loop, which may play a role in organizing the higher-order structure.

## Abstract 2

The same hexagonal array of archaerhodpsin-2 trimers as observed in the native membrane is reconstituted in a three-dimensional crystal prepared by the membrane fusion method. In this crystal, a pair of conserved glutamate residues in the proton-release pathway is maintained by a low-barrier hydrogen bond.

## 1. Introduction   

Since the light-driven proton pump bacteriorhodopsin was discovered in the cell membrane of *Halobacterium salinarum* (Oesterhelt & Stoeckenius, 1971 ▶), a large number of proton-pumping rhodopsins have been found in diverse species, including archaea, eubacteria, fungi and algae (Ihara *et al.*, 1999 ▶; Waschuk *et al.*, 2005 ▶; Spudich *et al.*, 2014 ▶). To date, nine proton-pumping rhodopsins [*i.e.* archaerhodopsin-1 and archaerhodopsin-2; (aR1 and aR2; Enami *et al.*, 2006 ▶; Yoshimura & Kouyama, 2008 ▶), acetabularia rhodopsin II (Wada *et al.*, 2011 ▶), bacteriorhodopsin (bR; Essen *et al.*, 1998 ▶; Pebay-Peyroula *et al.*, 1997 ▶; Sato *et al.*, 1999 ▶; Faham & Bowie, 2002 ▶), cruxrhodopsin-3 (cR3; Chan *et al.*, 2014 ▶), deltarhodopsin-3 (dR3; Zhang *et al.*, 2013 ▶), proteorhodopsin (Gushchin *et al.*, 2013 ▶; Ran *et al.*, 2013 ▶) and xanthorhodopsin (Luecke *et al.*, 2008 ▶)] have been crystallized and their structural data have enabled the discussion of a common structural motif that is relevant to the proton-pumping activity (Kouyama & Murakami, 2010 ▶; Ernst *et al.*, 2014 ▶).

**Figure 1:**
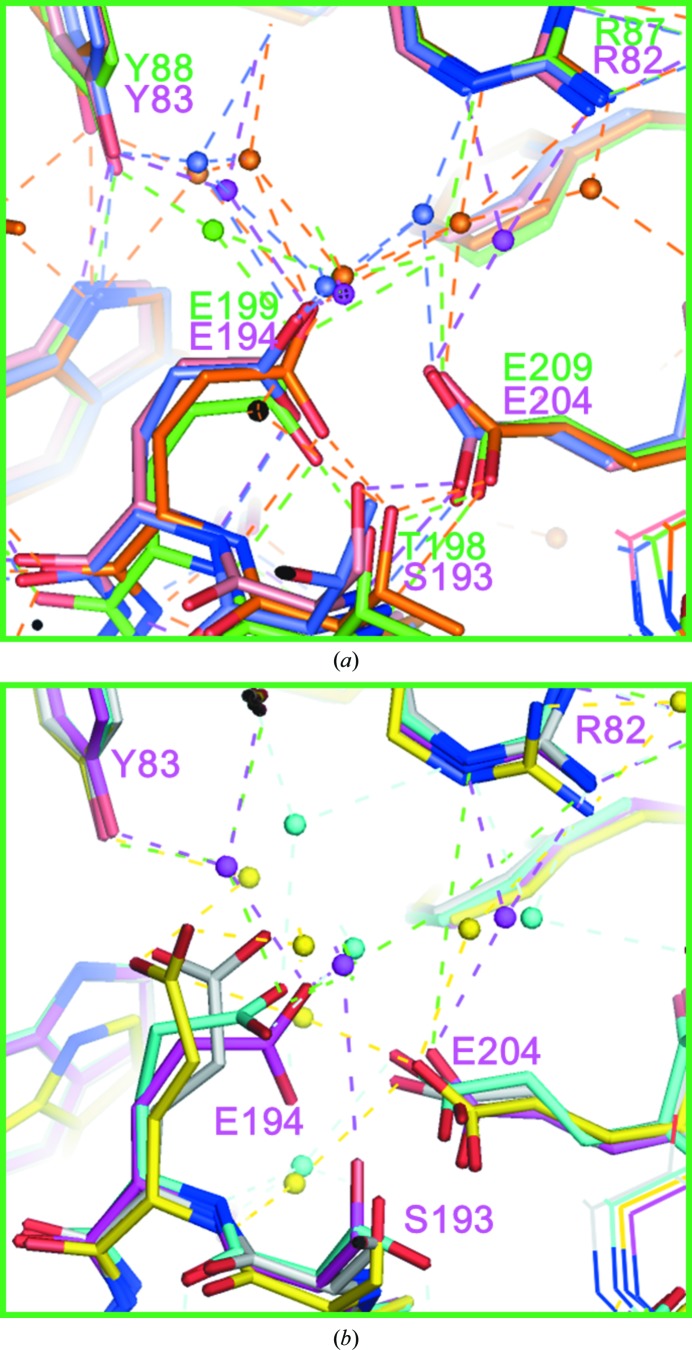
(*a*) The structure of the proton-release channel observed for the neutral purple form of bR in the *P*622 crystal (purple; PDB entry 1iw6; Matsui *et al.*, 2002 ▶) is compared with those observed for aR2 in the *P*321 crystal (green; PDB entry 2ei4; Yoshimura & Kouyama, 2008 ▶), cR3 in the *P*321 crystal (orange; PDB entry 4jr8; Chan *et al.*, 2014 ▶) and dR3 in the *R*32 crystal (blue; PDB entry 4fbz; Zhang *et al.*, 2013 ▶). (*b*) The structure of the proton-release channel observed for the neutral purple form of bR in the *P*622 crystal (purple) is compared with those observed for the alkaline pink form of bR in the *P*622 crystal (yellow; PDB entry 1xok; Okumura *et al.*, 2005 ▶), bR in the *C*2 crystal (PDB entry 1brr; Essen *et al.*, 1998 ▶) and bR in the *P*6_3_ crystal (PDB entry 1qhj; Belrhali *et al.*, 1999 ▶).

Interestingly, the diffraction data from three-dimensional crystals of bR, aR2, cR3 and dR3 that were prepared by the membrane-fusion method have shown that at neutral pH two glutamates in the proton-release channel form a paired structure (Fig. 1 ▶ *a*). On the basis of the pH dependence of the protein structure, it has been argued that the paired structure of Glu194 and Glu204 in bR is maintained by a low-barrier hydrogen bond (Okumura *et al.*, 2005 ▶). This argument has been supported by a recent theoretical study (Phatak *et al.*, 2008 ▶). Since the paired structure of Glu194 and Glu204 in bR is broken during the proton-pumping cycle, it has been suggested that the Glu194/Glu204 pair functions as a proton-release complex (Takeda *et al.*, 2004 ▶; Yamamoto *et al.*, 2009 ▶). On the other hand, structural models of bR determined using different crystal forms have suggested that the conformation of the proton-release channel is sensitive to the crystallization procedure (Fig. 1 ▶ *b*). In an orthorhombic crystal (space group *C*2) that was prepared by the epitaxial growth method, the side chain of Glu194^bR^ is directed towards Tyr83^bR^ (Essen *et al.*, 1998 ▶). In a *P*6_3_ crystal that was prepared by embedding the membrane protein in a cubic phase of monooleic acid, the side chain of Glu194^bR^ is hydrogen-bonded to Ser193^bR^ OH (Luecke, 2000 ▶). This configuration is distinct from that found in the *P*622 crystal of bR at neutral pH, in which Glu194^bR^ is hydrogen-bonded to the backbone amide NH of the same residue (Okumura *et al.*, 2005 ▶). Since the lipid component (for example, triglycolipid) observed in the *P*622 crystal is partially removed or missing in the other crystal forms (Belrhali *et al.*, 1999 ▶), it is possible that the conformation of the functionally important region is influenced by the lipid–protein inter­actions. Their influence cannot be ignored in discussion of the structural changes that occur during the proton-pumping cycle. For example, there is a significant difference among the reported structural models of the L state (Royant *et al.*, 2001 ▶; Kouyama *et al.*, 2004 ▶; Lanyi, 2005 ▶). For a better understanding of the proton-pumping mechanism, it is important to accumulate structural data on other proton-pumping rhodopsins using high-quality three-dimensional crystals.

Archaerhodopsin-2 (aR2) is a light-driven proton pump that is found in the cell membrane of *Halorubrum* sp. Aus-2 (Uegaki *et al.*, 1991 ▶). The sequence identity among members of the archaerhodopsin family is high (>90%; Mukohata *et al.*, 1991 ▶; Ihara *et al.*, 1999 ▶), whereas the amino-acid sequences of all members of the aR family are distinct from those of proton-pumping rhodopsins with known structure (*i.e.* the sequence identities are less than 50%). In previous studies, aR2 was crystallized as three-dimensional crystals belonging to space groups *C*222_1_ (Enami *et al.*, 2006 ▶), *P*321 and *P*6_3_ (Yoshimura & Kouyama, 2008 ▶). In the *P*321 and *P*6_3_ crystals aR2 assembles into a trimeric structure with bacterioruberin bound to the inter-subunit crevice. This trimeric structure is destroyed in the *C*222_1_ crystal. Comparison of the structures of aR2 in these crystal forms revealed that the conformation of the extracellular end of helix C is greatly altered upon removal of the lipid component interacting with helix C. It would be interesting to investigate how much the structures of the photoreaction intermediates are affected by the protein packing or the protein–lipid interactions. Unfortunately, the previously reported three-dimensional crystals of aR2 were merohedrally twinned, making it difficult to analyze the possible effects of light-induced structural changes in aR2.

In this study, we have applied the membrane-fusion method to prepare a novel three-dimensional crystal of aR2. This crystal is made up of membranous layers, in each of which the aR2 trimers are arranged on a hexagonal lattice. Compared with the previously reported crystal forms of aR2, the new crystal has several advantages: (i) it diffracts X-rays to a higher resolution, (ii) the problem of crystal twinning is avoided and (iii) the hexagonal array as seen in the native claret membrane is reconstituted in the crystal. Owing to these properties, the new crystal provided accurate structural information about functionally important residues. It is shown that two glutamates (Glu199 and Glu209) in the proton-release channel are separated by a very short distance (2.3 Å). This result suggests that their paired structure is maintained by a low-barrier hydrogen bond. Meanwhile, flash-induced absorption changes in aR2 showed that the K state decayed directly to the M state; *i.e.* the L state was undetected in the photocycle of aR2. On the basis of these newly obtained structural and absorption kinetics data for aR2, we discuss possible structural factors affecting the photoreaction kinetics and the higher-order structure of proton-pumping rhodopsin.

## 2. Materials and methods   

### 2.1. Preparation and crystallization of archaerhodopsin-2   

Claret membrane containing archaerhodopsin-2 was isolated from *Halorubrum* sp. Aus-2 and purified according to a previously described procedure (Enami *et al.*, 2006 ▶). For the crystallization of aR2, a mixture consisting of claret membrane (∼3 mg ml^−1^), 5 mg ml^−1^ nonylglucoside, 1 *M* ammonium sulfate, 0.08 *M* sodium chloride, 0.04 *M* sodium azide and 0.04 *M* HEPES pH 7 was slowly concentrated by the sitting-drop vapour-diffusion method using 0.5 ml 2.2–2.8 *M* ammonium sulfate, ∼0.1 *M* sodium citrate pH 7 as a reservoir solution. Incubation at 15°C for approximately one month yielded hexagonal rod crystals with typical dimensions of 50 × 50 × 200 µm.

### 2.2. Measurement of absorption kinetics   

Transient transmission data from aR2 in claret membranes were acquired using a frequency-doubled Nd-YAG laser as described previously (Hayakawa *et al.*, 2008 ▶). The absorption kinetics measured at various wavelengths were analyzed using the singular value decomposition method (Chizhov *et al.*, 1996 ▶).

### 2.3. Data collection, scaling and refinement   

For X-ray diffraction measurements, a single crystal was picked up and soaked in a post-crystallization solution consisting of 2.2 *M* ammonium sulfate, 0.1 *M* HEPES pH 7, 30% trehalose for ∼10 min; subsequently, the crystal was flash-cooled in dim light with liquid propane at its melting temperature. X-ray diffraction data were collected on beamline BL38B1 at SPring-8, where a crystal kept at 100 K was exposed to a monochromatic X-ray beam at a wavelength of 1.0 Å with an X-ray flux rate of ∼2 × 10^12^ photons mm^−2^ s^−1^. Diffraction data were collected using an oscillation range of 1° and an X-ray flux of ∼1 × 10^13^ photons mm^−2^ per image. Indexing and integration of diffraction spots were carried out using *MOSFLM* 6.1 (Steller *et al.*, 1997 ▶). The scaling of data was accomplished using *SCALA* in the *CCP*4 program suite (Winn *et al.*, 2011 ▶). Structural analysis was performed with *CNS* (Brünger *et al.*, 1998 ▶), *REFMAC*5 (Murshudov *et al.*, 2011 ▶) and *XtalView* (McRee, 1993 ▶). The structure of aR2 in the *P*321 crystal (PDB entry 2ei4) was used as an initial model. After rotational and translational searching with *MOLREP* (Vagin & Teplyakov, 2010 ▶), water and lipid molecules were added on the basis of the 2*F*_o_ − *F*_c_ map and the structure was refined by several cycles of simulated-annealing and individual *B*-factor refinements. The final refinement of the protein structure using *REFMAC*5 resulted in an *R*_cryst_ of 21.4% and an *R*_free_ of 23.4% (Table 1 ▶).

## 3. Results   

### 3.1. Crystal packing   

**Figure 2:**
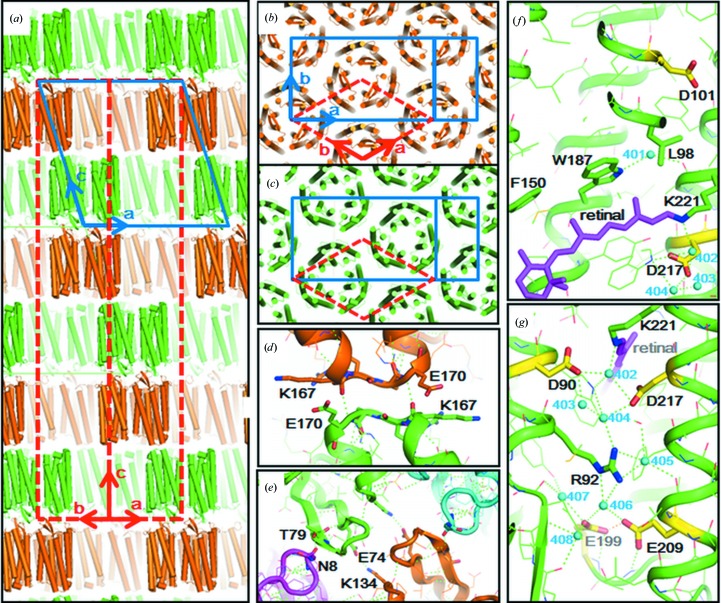
(*a*, *b*, *c*) Protein packing in the hexagonal crystal of aR2 used in this study. The protein arrangement is described by space group *H*32 (red broken lines) or *C*2 (solid blue lines). (*d*) Intermembrane protein–protein contacts on the cytoplasmic side. (*e*) Intermembrane protein–protein contacts on the cytoplasmic surface. (*f*, *g*) Distribution of water molecules (cyan spheres) in proton-uptake and proton-release channels.

**Figure 3:**
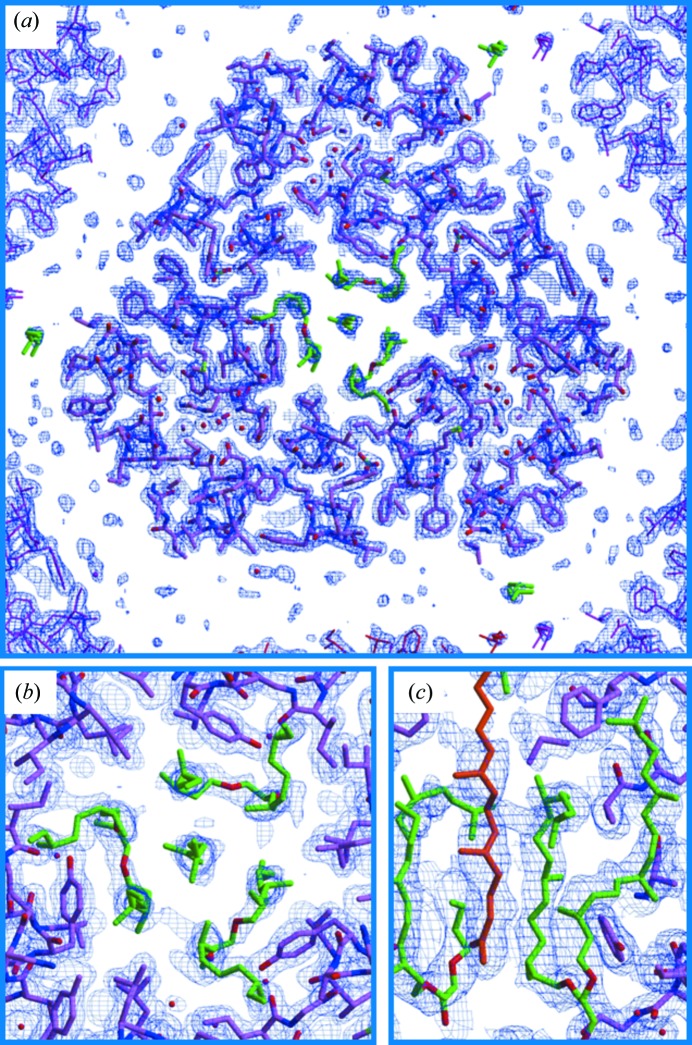
A 2*F*_o_ − *F*_c_ map contoured at 1.4σ (*a*) or 0.8σ (*b*, *c*) is superimposed on the structural model that was built in space group *C*2. Lipid molecules trapped in the central opening (the extracellular side) of the aR2 trimer are shown in green or brown.

In the first approach to model building, the crystal was presumed to belong to space group *C*2, with unit-cell parameters *a* = 108.6, *b* = 62.7, *c* = 116.3 Å, α = 90, β = 108.1, γ = 90° (blue solid lines in Figs. 2 ▶ *a* and 2 ▶ *b*). In this space group, the asymmetric unit contains three subunits of aR2 (*i.e.* one trimer). The side view of the unit cell shows that the crystal is composed of membranous layers (Fig. 2 ▶ *a*). In each membranous layer, aR2 trimers are arranged on a hexagonal lattice with a cell dimension of 62.7 Å (Fig. 2 ▶ *b*). At first sight, it appeared that there was no structural difference among the three protein molecules in the asymmetric unit, each of which was folded into seven transmembrane helices (helices A–G), an Ω-loop in the N-terminal region (Asn5–Pro12) and a β-sheet at the BC loop. However, close investigation of the electron-density map showed that the lipid molecules trapped within the aR2 trimer were not distributed with perfect threefold symmetry (Fig. 3 ▶). Specifically, a hydrocarbon chain (possibly squalene) is positioned at the centre of the trimer, breaking the threefold symmetry. Although its extracellular half with an extended conformation is surrounded by three diphytanyl chains, the cytoplasmic half is disordered. Disorder was also observed for other lipids filling the inter-trimer space and the side chains of arginine residues (Arg34 and Arg230) that can interact with negatively charged lipid head groups. Strictly speaking, we need to postulate that the three subunits in each trimer adopt slightly different conformations. However, the structural differences among these subunits would be difficult to analyze unless all of the trimers within a membranous layer have the same in-membrane orientation. In the current model building in space group *C*2 only the trimeric structure that is averaged over three possible in-membrane orientations can be analyzed.

**Figure 4:**
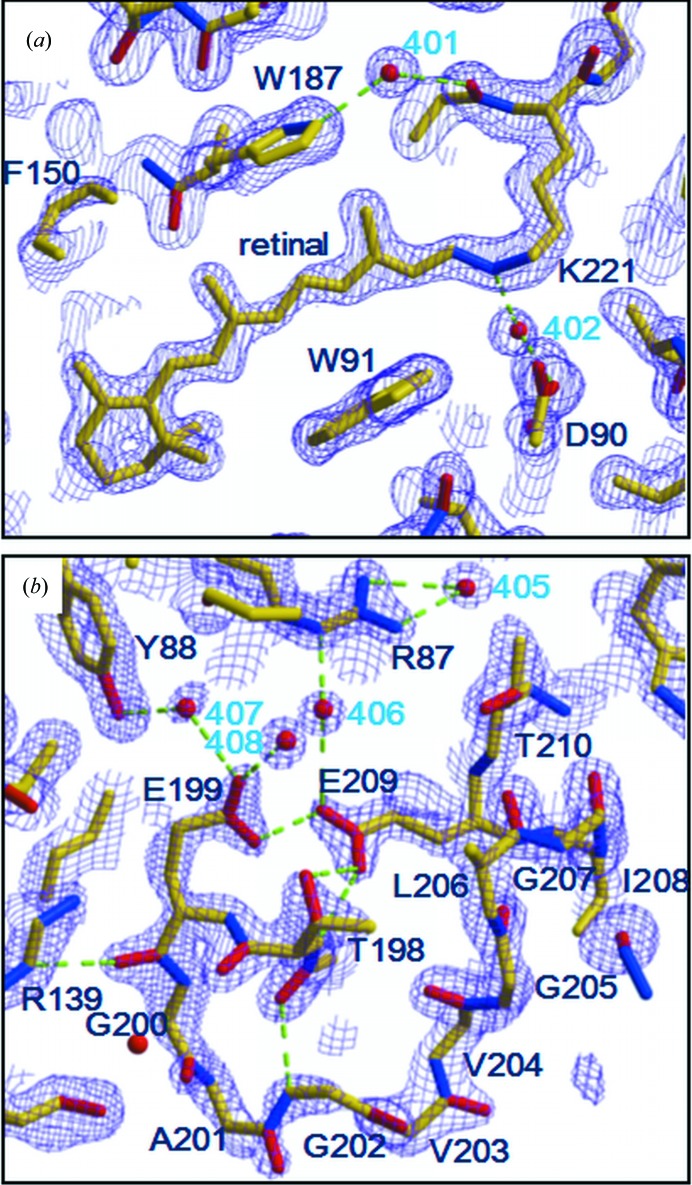
2*F*_o_ − *F*_c_ maps around the retinal chromophore (*a*) and the FG loop (*b*) contoured at 1.6σ are superimposed on the structural model that was built in space group *H*32.

In the second approach to model building, the crystal used in this study was assumed to belong to space group *H*32, with unit-cell parameters *a* = *b* = 62.7, *c *= 331.5 Å, α = 90, β = 90, γ = 120° (red broken lines in Figs. 2 ▶ *a*, 2 ▶ *b* and 2 ▶ *c*). In space group *H*32, one subunit of aR2 is contained in the asymmetric unit; *i.e.* the aR2 trimer is supposed to have perfect threefold symmetry. It should be pointed out that the structural model constructed on the assumption of space group *H*32 is equivalent to the averaged structure of the three subunits that are contained in the asymmetric unit of space group *C*2. Indeed, there was no essential difference between the structural models constructed by the above two approaches. In the following discussion, we describe the structural model built in space group *H*32, which provides more accurate structural information about the functionally important residues (Fig. 4 ▶). Hereafter, the hexagonal crystal investigated in this study will be called the *H*32 crystal.

**Figure 5:**
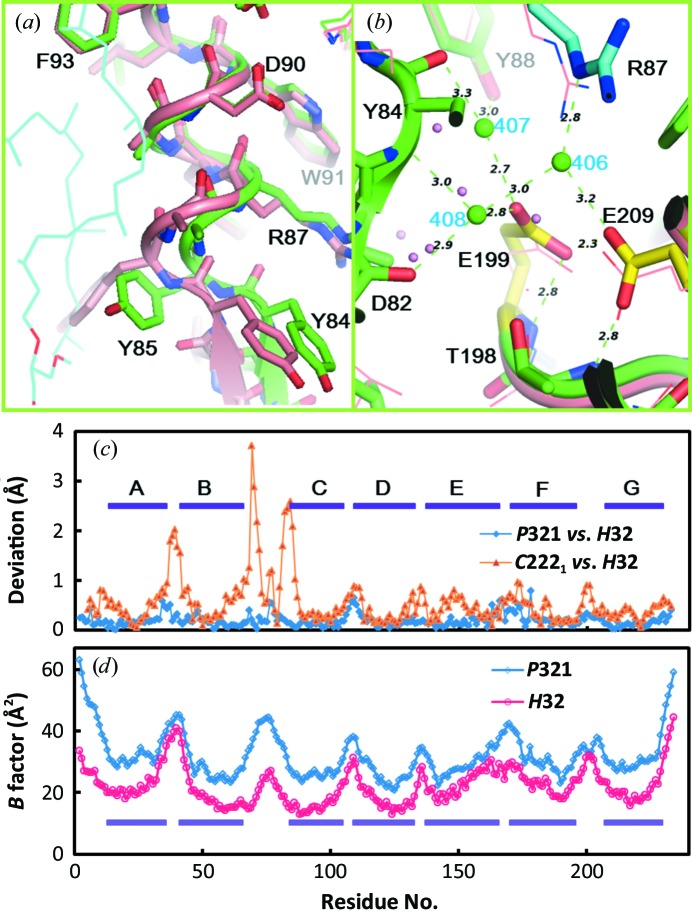
The structures of the extracellular half of helix C (*a*) and the proton-release channel (*b*) of aR2 in the *H*32 crystal (purple, yellow and cyan) are compared with those observed in the *C*222_1_ crystal (salmon; PDB entry 1vgo; Enami *et al.*, 2006 ▶). (*c*) Structural differences between the *H*32 crystal and the *P*321 crystal (PDB entry 2ei4; blue; Yoshimura & Kouyama, 2008 ▶) and between the *H*32 crystal and the *C*222_1_ crystal (orange). The deviation of the C^α^ atom is plotted against the residue number. (*d*) The *B* factors observed in the *H*32 crystal (magenta) and the *P*321 crystal (blue). The *B* factor of the C^α^ atom is plotted against the residue number.

Since the membranous layers in the *H*32 crystal are piled up with alternate orientations, there are two types of intermembrane protein–protein contacts. On the extracellular side, the BC loop interacts with the DE loop of one of the neighbouring proteins and the N-terminal region of another neighbouring protein (Fig. 2 ▶ *d*). On the cytoplasmic side, the EF loop contacts the EF loop of the protein opposite (Fig. 2 ▶ *e*). The structure of the protein–protein contact region is slightly different from that observed in the *P*321 crystal, in which the aR trimers are piled up directly along the *c* axis (Yoshimura & Kouyama, 2008 ▶). A more significant difference is that the *B* factors of the main-chain atoms in the extracellular halves of helices A, B, C and D are much lower in the *H*32 crystal than in the *P*321 crystal (Fig. 5 ▶ *d*). It is suggested that the motional freedom of these regions is suppressed by the inter-membrane protein–protein contacts in the *H*32 crystal. On the other hand, relatively higher *B* factors are observed for the main-chain atoms in the cytoplasmic halves of helices E and F. This result suggests that the intermembrane protein–protein interaction at the EF loop is weak. It is unlikely that this interaction is a major factor in determining the cell dimension of the hexagonal lattice.

It has been reported that in freshly prepared claret membrane the aR2 trimers are arranged in a hexagonal lattice with a cell dimension of 63.3 Å at room temperature (Yoshimura & Kouyama, 2008 ▶). This cell dimension is nearly identical to that (62.7 Å) of the hexagonal lattice found in the *H*32 crystal. (The small difference may be owing to shrinkage of the hexagonal lattice at the cryogenic temperature.) Thus, it appears possible that successive fusion of native claret membranes in the pre-crystallization solution yields flat sac-like structures that are then piled up in the crystal-growth phase.

In many respects, the membranous structure found in the *H*32 crystal resembles the purple membrane of *H. salinarum*. Firstly, the centre-to-centre distance (62.7 Å) between neighbouring aR2 trimers is similar to the cell dimension (62.45 Å) of the hexagonal lattice in the purple membrane (Grigorieff *et al.*, 1996 ▶). Secondly, the in-membrane orientation of the aR2 trimer is identical to that of the bR trimer in the purple membrane. This similarity can be explained by taking into account the outline of the trimeric structure on the extracellular side, which is approximated by a truncated triangle. Since there is no direct protein–protein interaction between neighbouring trimers in the same membrane (Fig. 3 ▶), it is rational to suppose that the lipid molecules filling the inter-trimer space play an important role in maintaining the two-dimensional hexagonal arrays of the aR2 or bR trimer. It was observed, however, that most of the lipid molecules filling the inter-trimer space were apparently disordered. One possible explanation for such disorder is that claret membrane contains a special lipid component that destroys the local threefold symmetry. Such a lipid distribution may be effective at creating a flexible lipid environment that may be necessary for the protein to undergo large structural changes during the proton-pumping cycle.

### 3.2. Structure of the proton-release channel   

The high-resolution diffraction data from the *H*32 crystal allowed us to accurately determine the conformations of two glutamates (Glu199 and Glu209) present in the proton-release channel (Fig. 2 ▶ *g*). When the structural model was refined using *REFMAC*5, the distance between Glu199 OE2 and Glu209 OE1 was determined to be 2.3 Å (Fig. 5 ▶ *b*). (A slightly longer distance was evaluated when the structure was refined by *CNS* with default parameters.) This short distance suggests that the paired structure of Glu199 and Glu209 is maintained by a low-barrier hydrogen bond. Glu199 OE2 is directed towards the main-chain amide NH of the same residue. This conformation of Glu199 is nearly identical to that observed in the *P*321 or *P*6_3_ crystal (Fig. 1 ▶ *a*). When the protein structure in the *H*32 crystal is compared with that found in the *C*222_1_ crystal, however, a significant difference is observed in the proton-release channel (Figs. 5 ▶ *a* and 5 ▶ *b*). Namely, the extracellular end of helix C moves outwards when the lipid distribution around the protein is disturbed during the crystallization process of the *C*222_1_ crystal. It appears that the proton-release channel takes on a closed conformation as long as the extracellular end of helix C interacts with the diphytanyl group of the native lipid that fills the central opening of the aR2 trimer.

### 3.3. Structure of the retinal-binding pocket   

**Figure 6:**
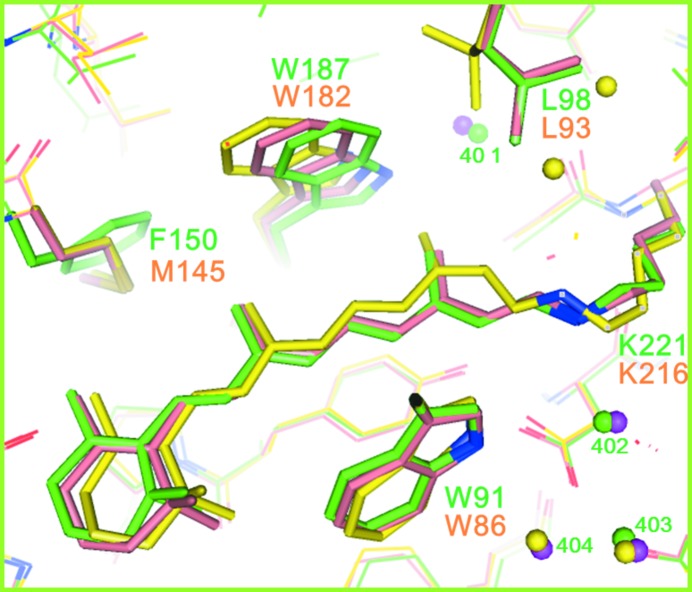
The structure of aR2 in the *H*32 crystal (green) is compared with those of the unphotolyzed state (salmon; PDB entry 1iw6; Matsui *et al.*, 2002 ▶) and the L state (yellow; PDB entry 1ucq; Kouyama *et al.*, 2004 ▶) of bR.

The retinal chromophore bound to the ∊-amino group of Lys220^aR2^ in helix G adopts the all-*trans* configuration. Although most residues in the retinal-binding pocket are highly conserved in proton-pumping archaeal rhodopsins, Met145 in bR is replaced by phenylalanine (Phe150) in aR2. Owing to this replacement, the polyene chain of retinal adopts a less curved conformation in aR2 than in bR (Fig. 6 ▶). This difference can explain the large blue shift in the visible absorption band of retinal (λ_max_ ≃ 550 nm in aR2 *versus* λ_max_ ≃ 570 nm in bR).

Another effect of the Met145^bR^→Phe150^aR2^ replacement is seen as a noticeable shift in the indole ring of the tryptophan residue (Trp182^bR^, Trp187^aR2^) in contact with the C13 methyl group of retinal towards helix G. Previous structural analysis of the K and L states of bR have shown that the relaxation of retinal into a planar 13-*cis* configuration in the K-to-L transition is accompanied by a noticeable movement in the indole ring of Trp182^bR^ towards Met145^bR^ and a large rotation of the side chain of Leu93^bR^ (Fig. 6 ▶, model shown in yellow; Matsui *et al.*, 2002 ▶; Kouyama *et al.*, 2004 ▶). It is noteworthy that in aR2 the motional freedom of the tryptophan residue (Trp187^aR2^) in contact with the C13 methyl of retinal is reduced by the Met145^bR^→Phe150^aR2^ replacement. Thus, it would be expected that the K-to-L transition is slowed down in aR2. To examine this possibility, we measured the absorption kinetics of aR2 under various solvent conditions.

**Figure 7:**
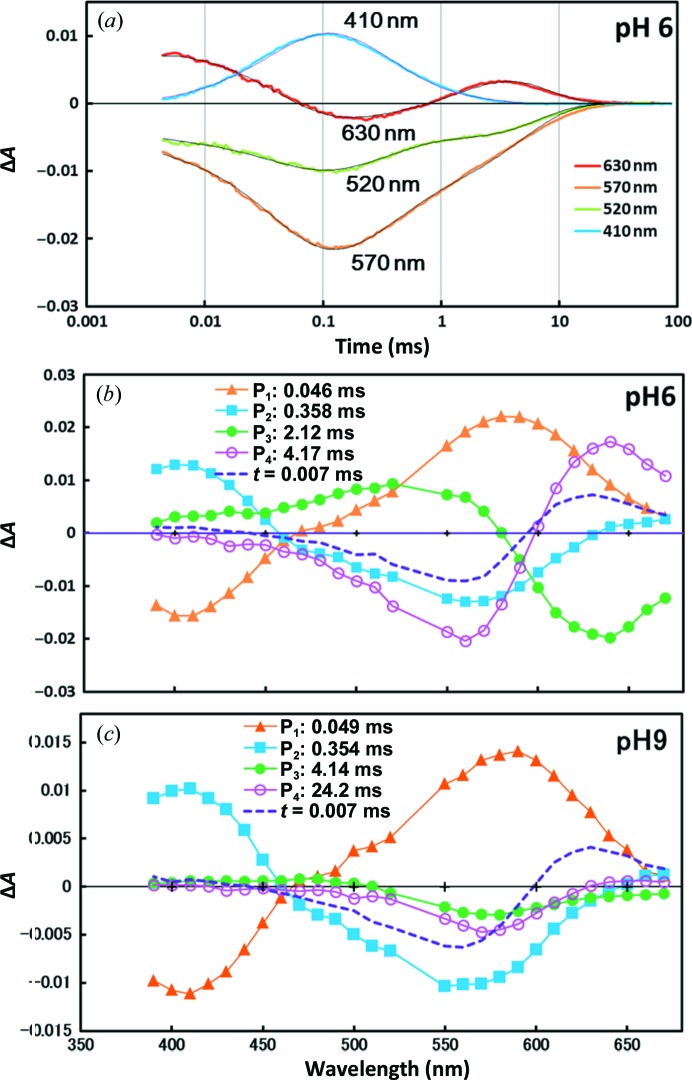
Flash-induced absorption changes in aR2 at pH 6 (*a*, *b*) and pH 9 (*c*). (*a*) An aqueous suspension of claret membranes was excited with light pulses at 532 nm and absorption changes were recorded at various wavelengths. (*b*, *c*) The absorption kinetics in the time range from 0.003  to 90 ms were fitted with four components and the amplitude of each component was plotted against the wavelength of the light used for measurement. The dashed line represents the absorption change observed ∼7 µs after the excitation.

Fig. 7 ▶ shows the flash-induced absorption changes in aR2 observed in an aqueous suspension of claret membrane. At pH 6, the absorption kinetics data were fitted with four exponential components. The difference spectrum associated with the P_1_ component exhibits a positive peak at 580 nm and a negative peak at 410 nm, suggesting that the K state decays directly into the M state. This transition is followed by the M-to-N transition (the P_2_ component with a positive peak at 410 nm and a negative peak around 560 nm), the N-to-O transition (the P_3_ component with a positive peak at 520 nm and a negative peak at 640 nm) and the process of recovering the initial state (the P_4_ component with a positive peak at 640 nm and a negative peak at 560 nm). At a higher pH (*e.g.* at pH 9) the decay rate of N became lower, making it difficult to separately describe the decay kinetics of the N and O states. Meanwhile, the decay kinetics of the K and M states remained unaltered over a wide pH range (pH 5–9). Under the investigated solvent conditions, the photocycle of aR2 can be described by the scheme K→M→N→O→aR2.

Compared with the photocycle of bR (K→L→M→N→O→bR; Lozier *et al.*, 1992 ▶), the photoreaction of aR2 is peculiar in that the L state is not detected. It is noteworthy that the lifetime of the K state of aR2 (∼40 µs) is much longer than that observed in bR (∼1 µs). It is probable that the L state is undetectable because the decay rate of L is much higher than the decay rate of K. Together with the crystallographic data, the absorption kinetics data suggest that the L state is destabilized when the structure in the cytoplasmic vicinity of retinal is made rigid.

### 3.4. Structure of the N-terminal polypeptide   

**Figure 8:**
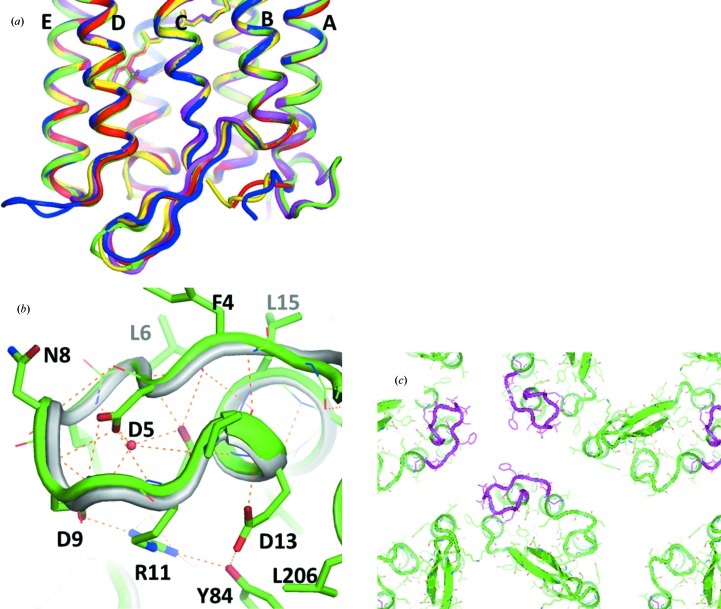
(*a*) Structural comparison of aR2 in the *H*32 crystal (green), bR in the *P*622 crystal (orange; PDB entry 1iw6; Matsui *et al.*, 2002 ▶), cR3 in the *P*321 crystal (blue; PDB entry 4jr8; Chan *et al.*, 2014 ▶) and dR3 in the *R*32 crystal (yellow; PDB entry 4fbz; Zhang *et al.*, 2013 ▶). (*b*) The N-terminal region of aR2 in the *H*32 crystal (green) is compared with that of aR1 in the *P*4_3_2_1_2 crystal (grey; PDB entry 1uaz; Enami *et al.*, 2003 ▶). (*c*) The outline of the inter-trimer space in the hexagonal lattice found in the *H*32 crystal of aR2, viewed from the extracellular side. The N-terminal region of aR2 is shown in magenta.

Compared with other proton-pumping rhodopsins, the structure of aR2 is peculiar in that its N-terminal polypeptide (Phe4–Pro12) folds into an Ω-loop (Fig. 8 ▶ *b*). This loop structure is maintained by hydrogen bonds between the side chain of Asp5 and the main-chain amide NH atoms of three residues (Asn8, Asp9 and Gly10; Fig. 8 ▶ *b*). The side chain of Arg11 (and Glu13 at the extracellular end of helix A) interacts with Tyr84, which is located at the cytoplasmic end of helix C. This interaction contributes to fixation of the Ω-loop.

The structure of the N-terminal polypeptide in the *H*32 crystal is nearly identical to that observed in the *P*321 or *P*6_3_ crystals, in which the aR2 trimers are packed in different fashions (Yoshimura & Kouyama, 2008 ▶). This suggests that the structure of the Ω-loop is strongly built, although the *B* factors of the main-chain atoms are high unless the wobbling motion of the Ω-loop is suppressed by protein–protein interactions. It is noteworthy that the amino-acid sequence in the Ω-loop is highly conserved among members of the archaerhodopsin family (Mukohata *et al.*, 1991 ▶; Ihara *et al.*, 1999 ▶). Indeed, the N-terminal region of aR1 folds into the same Ω-loop as observed in aR2 (Enami *et al.*, 2006 ▶). Since this loop interacts with the extracellular end of helix C, it seems possible that this loop may play a role in stabilizing the native conformation of the proton-release channel.

It has previously been proposed that the segregation of membrane proteins in biological membranes can be explained by a hydrophobic matching principle related to the difference in lipid bilayer hydrophobic thickness and protein hydrophobic length (Sabra & Mouritsen, 1998 ▶). It is noteworthy that in the *H*32 crystal the side chains of hydrophobic residues (Phe4 and Leu6) in the N-terminal region of aR2 are directed towards a crystallographic threefold axis (Fig. 8 ▶). These residues contribute to a substantial increase in protein hydrophobic length. This peculiar structural feature may confer aR2 with a high ability to assemble into a two-dimensional crystalline array. Indeed, it was observed that aR2-containing claret membrane was reluctant to disassemble in the presence of 50 m*M* nonylglucoside at 30°C, whereas the purple membrane of *H. salinarum* was completely solubilized under the same condition.

## 4. Discussion   

It is shown here that two glutamates (Glu199^aR2^ and Glu209^aR2^) in the proton-release channel form a paired structure that is maintained by a low-barrier hydrogen bond. This same conformation of the proton-release complex has been observed in the *P*622 crystal of bR, the *P*321 crystal of cruxrhodopsin-3 and the *R*32 crystal of deltarhodopsin-3, all of which were prepared by the membrane-fusion method (Okumura *et al.*, 2005 ▶; Yoshimura & Kouyama, 2008 ▶; Zhang *et al.*, 2013 ▶; Chan *et al.*, 2014 ▶). It has been reported that the *P*622 crystal of bR has an absorption peak at 568 nm in the neutral pH range (pH 4–9), whereas alkalization above pH 9.5 causes a noticeable blue shift of the visible absorption band (Okumura *et al.*, 2005 ▶). Diffraction data from the *P*622 crystal soaked at high pH levels have shown that the alkaline transition is accompanied by reorientation of the carboxyl group of Glu194^bR^ towards Tyr83^bR^ (Fig. 1 ▶ *b*). It is interesting to note that the conformation of Glu194^bR^ in the alkaline pink conformer is very similar to that observed in the *C*2 crystal of bR (Fig. 1 ▶ *b*). This similarity suggests that the transition to the alkaline pink conformer occurs at a much lower pH in the *C*2 crystal than in the *P*622 crystal. It is conceivable that the neutral purple form of bR is destabilized when the local pH at the membrane surface is substantially increased by the partial removal of negatively charged native lipids. In fact, the lipid component (for example triglycolipid) observed in the *P*622 crystal is partially removed or missing in other crystal forms. It has been reported that the neutral purple form is destabilized in bR-containing vesicles reconstituted with egg lethicin, in which the transition from the neutral purple form into an inactive red form with λ_max_ at 480 nm takes place at pH 6.5 (Nasuda-Kouyama *et al.*, 1990 ▶).

With respect to the protein structure in the *P*6_3_ crystal, Glu194 OE2 in the structural model solved at 1.55 Å resolution (PDB entry 1c3w) has an unusually large *B* factor (62 Å^2^; Luecke *et al.*, 1999 ▶). (The *B* factor of Glu199 OE2 of aR2 in the *H*32 crystal is 31 Å^2^.) It is possible that the conformation of Glu194^bR^ in the *P*6_3_ crystal represents a mixed state between the neutral purple form and the alkaline pink form. There is another caveat to the structural model of bR that was built using the *P*6_3_ crystal. Although the model building was performed on the supposition that the lipid molecules filling the intra-trimer or inter-trimer space are distributed with threefold symmetry, this supposition may not be correct. In fact, the lipid head groups are not included in any reported model of bR determined using the *P*6_3_ crystal. For example, three triglycolipids are shown to bind to the central opening of the bR trimer in the *P*622 crystal, whereas this lipid component is missing in the *P*6_3_ crystal. Since a step of protein solubilization is included in the preparation procedure of the *P*6_3_ crystal, it is likely that the lipid distribution around the protein is altered at a pre-crystallization stage.

If the Glu194/Glu204 paired structure is already broken in the unphotolyzed state, the energetic barrier to a light-induced structural change in the proton-release channel would be lowered. It has been reported that the rising rate of the M state of bR is much higher in the *P*6_3_ crystal than in native purple membrane or the *P*622 crystal (Royant *et al.*, 2001 ▶; Yamamoto *et al.*, 2009 ▶). Therefore, it is not surprising to find that the L state generated in the *P*6_3_ crystal is structurally different from that observed in the *P*622 crystal (Royant *et al.*, 2000 ▶; Kouyama *et al.*, 2004 ▶; Lanyi & Schobert, 2003 ▶). For example, a large swing of the side chain of Arg82 towards Glu194 is seen in the former model, whereas this swing is not seen in the latter model. Instead, the K-to-L transition in the *P*622 crystal is accompanied by a large structural change in the cytoplasmic vicinity of retinal, including a noticeable movement of the indole ring of Trp182^bR^ towards Met145^bR^. This light-induced structural change is in line with the present observation that the L state is destabilized when the motional freedom of the tryptophan in contact with the C13 methyl group is reduced by the Met145^bR^→Phe150^aR2^ replacement (Fig. 6 ▶).

## Supplementary Material

PDB reference: archaerhodopsin-2, 3wqj

## Figures and Tables

**Table 1:** Data-collection and final refinement statistics

Data collection		
Space group	*C*2	*H*32
Resolution (Å)	45.1–1.8 (1.85–1.80)	45.1–1.8 (1.90–1.80)
Unit-cell parameters		
*a* (Å)	108.6	62.74
*b* (Å)	62.7	62.74
*c* (Å)	116.3	331.45
α (°)	90.0	90.0
β (°)	108.1	90.0
γ (°)	90.0	120.0
No. of unique reflections	62577 (10063)	21944 (3462)
Multiplicity	3.2 (3.2)	8.8 (9.0)
Data completeness (%)	90.7 (100)	91.3 (100)
*R*_merge_ † (%)	4.6 (45.1)	5.4 (50.0)
〈*I*/σ(*I*)〉	17.3 (3.2)	28.5 (5.1)
Mosaicity (°)	0.50	0.48
Refinement		
Resolution limit (Å)	15.0–1.8	15.0–1.8
Protein residues	234 × 3	1–234
No. of lipids	13	5
No. of waters	155	51
*R*_cryst_ ‡ (%)	20.4	21.1
*R*_free_ (%)	22.7	24.1
R.m.s.d.		
Bond lengths (Å)	0.019	0.018
Bond angles (°)	1.95	2.03
*B* factor (Å^2^)		
Protein	23.5	23.5
Water	36.7	36.9
Lipids	58.6	60.2

† *R*_merge_ = 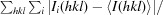 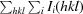, where *I*_*i*_(*hkl*) is the intensity of an individual reflection and 〈*I*(*hkl*)〉 is the mean intensity obtained from multiple observations of symmetry-related reflections.

‡ *R*_cryst_ = 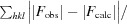 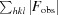. A randomly omitted 5% of the reflections were used for the calculation of *R*_free_.
